# Anti-nuclear autoantibodies in the general German population: prevalence and lack of association with selected cardiovascular and metabolic disorders—findings of a multicenter population-based study

**DOI:** 10.1186/s13075-017-1338-5

**Published:** 2017-06-06

**Authors:** Manas K. Akmatov, Nadja Röber, Wolfgang Ahrens, Dieter Flesch-Janys, Julia Fricke, Halina Greiser, Kathrin Günther, Rudolf Kaaks, Yvonne Kemmling, Bastian Krone, Jakob Linseisen, Christa Meisinger, Susanne Moebus, Nadia Obi, Carlos A. Guzman, Karsten Conrad, Frank Pessler

**Affiliations:** 1 TWINCORE, Centre for Experimental and Clinical Infection Research, Feodor-Lynen-Straße 7, 30625 Hannover, Germany; 2 Helmholtz Centre for Infection Research, Braunschweig, Germany; 3 Institute of Immunology, Technical University Dresden, Dresden, Germany; 4 Leibniz Institute for Prevention Research and Epidemiology—BIPS, Bremen, Germany; 5 University Medical Center Hamburg-Eppendorf, Hamburg, Germany; 6 German Cancer Research Center (DKFZ), Heidelberg, Germany; 7 Department of Epidemiology, Helmholtz Centre for Infection Research, Braunschweig, Germany; 8 Institute for Medical Informatics, Biometry and Epidemiology (IMIBE), University Clinics of Essen, Essen, Germany; 9 Helmholtz Zentrum München, Institute for Epidemiology II, Neuherberg, Germany; 10 Institute for Social Medicine, Epidemiology and Health Economics, Charité-Universitätsmedizin Berlin, Berlin, Germany; 11 Department of Vaccinology and Applied Microbiology, Helmholtz Centre for Infection Research, Braunschweig, Germany

**Keywords:** Anti-nuclear autoantibodies, Diabetes, German National Cohort, Hypertension, Metabolism, Obesity, Population-based study

## Abstract

**Background:**

We determined the prevalence of anti-nuclear autoantibodies (ANAs) in the German adult population and examined the association between ANAs and cardiovascular and metabolic disorders.

**Methods:**

We used data and blood samples from the pretest phases of the German National Cohort, obtained from six of the 18 study centers (*n* = 1199). All centers applied standardized instruments including face-to-face interviews, anthropometric measurements and collection of blood samples. Self-reported histories of diabetes mellitus, heart attack and elevated blood cholesterol and/or lipids were recorded. Height, weight and blood pressure were measured. ANAs were detected using a semi-automated system (AKLIDES®; Medipan GmbH, Dahlewitz, Germany). A positive ANA was defined as a titer ≥ 1:80. ANA were classified as weakly (1:80 or 1:160), moderately (1:320 or 1:640) or strongly (≥1:1280) positive. Specific autoantibodies against nuclear antigens were detected with second-step assays according to the ANA staining pattern. Associations between the assessed disorders and ANA positivity and pattern were examined using sex and age-adjusted mixed-effects logistic regression models.

**Results:**

Thirty-three percent (95% confidence interval; 31–36%) of the 1196 participants (measurements could not be obtained from three samples) were ANA positive (titer ≥ 1:80). The proportions of weakly, moderately and strongly positive ANA were 29%, 3.3% and 1.3%, respectively. ANA positivity was more common among women than men across all titers (χ^2^, *p* = 0.03). ANA positivity, even when stratified according to height of titer or immunofluorescent pattern, was not associated with diabetes, elevated blood cholesterol and/or lipids, obesity or hypertension. Second-step autoantibody assays were positive in 41 of the 83 samples (49%) tested, with anti-DFS70 (*n* = 13) and anti-dsDNA (*n* = 7) being most frequent. These subgroups were too small to test for associations with the disorders assessed.

**Conclusions:**

The prevalence of ANA positivity in the German general population was similar to values reported from other countries. Contrary to other studies, there was no association with selected self-reported and objectively measured cardiovascular and metabolic variables.

## Background

Anti-nuclear autoantibodies (ANAs) are immunoglobulins commonly used as an initial test to screen for connective tissue diseases such as systemic lupus erythematosus (SLE), systemic sclerosis, polymyositis, dermatomyositis or Sjögren’s syndrome (SjS) [1]. The ANA diagnostic is a sensitive test to evaluate these diseases, particularly SLE. However, the presence of ANAs is not specific for any particular connective tissue disease and can be associated with various other conditions such as cancer, chronic infections and cardiovascular diseases, and with use of certain medications [2, 3]. Even all-cause mortality has been associated with a positive ANA test [4]. One possible explanation for this phenomenon is that the presence of ANAs may reflect an increased baseline level of general inflammation and/or autoimmunity that is deleterious to the function of more than one organ. Tests for ANAs can also be positive in healthy individuals, particularly in low titers. For instance, a population-based study in China reported that approximately 6% of healthy individuals tested positive for ANA with a titer of 1:320 [5]. In a Mexican study, ANAs were detected in 35% of healthy individuals (titer 1:40) [6]. A higher risk of ANA positivity is associated with female sex [7] and increasing age [5]. Evidence is emerging that associations may also exist between ANA positivity and certain parameters of cardiovascular and metabolic dysfunction. For instance, high ANA titers have been associated with coronary atherosclerosis [8], and individuals positive for ANA were reported to be more likely to develop myocardial infarction and peripheral vascular disease [9]. In terms of metabolic endpoints, Gonzalez et al. [10] found an inverse association between obesity and ANA positivity in women (but none in men), and Heras et al. [11] observed higher ANA positivity among individuals with type 1 diabetes than among nondiabetic individuals. To our knowledge, there are no data derived from the German general population on ANA prevalence or the association between ANA positivity and cardiovascular and metabolic disorders. The aims of the present study were therefore to determine the prevalence of ANAs in a multicenter population-based study in Germany and to examine their association with selected cardiovascular and metabolic disorders.

## Methods

### Sampling

The German National Cohort (GNC, in German also known as the NaKo Gesundheitsstudie) is a large-scale multicenter population-based prospective cohort study aiming to recruit 200,000 male and female participants between 20 and 69 years of age in 18 study centers distributed across Germany [12]. Recruitment of participants for the main study started in 2014. For the present study, we used data and blood samples from the first and second pretest studies of the GNC conducted in 2011 and 2012, respectively, and covering six study centers (Augsburg, Bremen, Essen, Hamburg, Hannover and Heidelberg). The aim of the pretest studies was to test the feasibility of selected instruments. Population-based sampling was used to recruit participants, and therefore samples were drawn from population registries of the respective municipalities. In addition, in two study centers (Essen and Heidelberg), selected migrant populations were recruited using register and community-based approaches [13]. The latter included recruitment via social networks (e.g., in groceries frequented by migrants, mosques or general practitioners’ offices). The study population in Essen comprised individuals of Turkish origin only; in Heidelberg, in addition to nonmigrant individuals, recruitment included the two largest migrant population groups—that is, individuals of Turkish origin and ethnic German immigrants from the former Soviet Union (FSU resettlers) [13]. In both study centers, bilingual study documents (e.g., flyers, posters, questionnaires, etc.) were offered.

The proportion of older individuals was oversampled in that 26.7% were recruited in each of the three older age groups (40–49, 50–59 and 60–69 years) and 10% in each of the younger age groups (20–29 and 30–39 years). Individuals were contacted through land mail; nonresponders received up to two reminders and up to 10 telephone calls (provided that telephone numbers could be identified). Computer-assisted face-to-face interviews were performed to collect sociodemographic and health-related data in all study centers except Essen, where a questionnaire was administered. Anthropometric measurements were obtained from all participants. Finally, biologic specimens (e.g., blood, urine and stool samples, nasal swabs) were collected.

### Outcome variables

As part of the medical history, information on chronic diseases and medical events relevant to the presented study was collected by the questions “Have you ever been diagnosed with … [diabetes mellitus, myocardial infarction, elevated blood cholesterol and/or lipids] by a physician?”, with separate “yes”, “no” and “I don’t know” options for each item. Height and body weight were measured with the SECA 285 measuring station (SECA, Hamburg, Germany). Body mass index (BMI) was calculated using the formula weight/height^2^ (kg/m^2^). BMI ≥ 25 kg/m^2^ and BMI ≥ 30 kg/m^2^ were used to define overweight and obesity, respectively [14]. Blood pressure was measured three times within a 15-minute period with an HEM 705 IT blood pressure monitor (OMRON Healthcare Europe, the Netherlands), except in Essen where it was measured only once. We calculated the mean of the second and third measurements and defined hypertension as a systolic or diastolic blood pressure higher than 140 mmHg and/or 90 mmHg, respectively [15]. The study center in Essen was excluded from this analysis because only a single measurement was available. Self-reported information on hypertension was not used in our analysis.

### ANA as exposure variable

First, we divided the participants into two groups; negative and positive for ANA. A titer of at least 1:80 was used to define positivity. Second, participants were divided into four ANA subgroups: negative, weakly positive (titer 1:80 or 1:160), moderately positive (titer 1:320 or 1:640) and strongly positive (titer ≥1:1280).

### Laboratory analyses

ANAs were detected by indirect immunofluorescence on HEp-2 cells. The assessment of autoantibody titers and five main patterns (granular/fine granular, homogeneous/homogeneous fine granular, nucleolar, centromer and other patterns) was carried out with a semi-automated system (AKLIDES®; Medipan GmbH, Dahlewitz, Germany) [16–18] and confirmed by visual observation. Sera assessed as positive (titer ≥ 1:80) were further analyzed by specific second-step autoantibody assays according to the staining pattern. The selection of the following confirmatory assays was carried out according to test algorithms of routine diagnostics. For sera with granular/fine granular pattern, antibodies against Ro/SS-A, La/SS-B, U1-RNP and Sm were determined by ELISAs (Orgentec Diagnostika GmbH, Germany). Furthermore, immunodiffusion with extractable nuclear antigen (Hiss diagnostics GmbH, Germany) was applied. If homogeneous or homogeneous fine granular pattern occurred, ELISAs for the detection of antibodies against dsDNA, histones and nucleosomes (Seramun diagnostica GmbH, Germany) and Bioflash® chemiluminescence assay for the determination of DFS-70 antibodies (INOVA diagnostics Inc., USA) were performed. If a nucleolar pattern was observed, the EUROLINE® Immunoblot (EUROIMMUN Medizinische Labordiagnostika AG, Germany) for the detection of PMScl and Scl-70 antibodies was carried out. Because anti-centromere antibodies provide a specific pattern on HEp-2-cells, no antigen-specific confirmatory assay was necessary.

### Statistical analysis

Initially, we performed a descriptive analysis by study center. The differences in sociodemographic variables across the study centers were examined with the chi-square test or Fisher’s exact test. Furthermore, we pooled the data from all study centers and estimated the sex and age-specific prevalence of ANAs. The differences in ANA positivity between sex and country of birth were tested with the chi-square test or Fisher’s exact test. As a next step, we applied mixed-effects logistic regression analysis to examine the association between ANA positivity and cardiovascular and metabolic disorders. The models were adjusted for sex and age; the study center was included as a random effect. The procedure PROC GLIMMIX was used for mixed-effects logistic regression analysis. For this analysis, moderately and strongly positive samples were combined into one category because the proportion of strongly positive samples was very low to be analyzed separately. The analysis was performed with the statistical program SAS for Windows, version 9.2 (SAS Institute Inc., Cary, NC, USA) and R Foundation for Statistical Computing software (version 3.0.2).

## Results

### Description of the study population

Table 1 presents selected demographic and clinical data and ANA positivity by study center and for the total study population. The proportion of women was higher in all study centers, with the greatest differences in Essen and Heidelberg. The proportion of obese participants was twice as high in Essen as in the other study centers. There were apparent, albeit not statistically significant, differences across centers in the proportions of self-reported heart attacks (Fisher’s exact test, *p* = 0.18) or diabetes (χ^2^ = 5.129, df = 5, *p* = 0.40; Fisher’s exact test, *p* = 0.19).

**Table 1:** Sociodemographic characteristics, morbidities and ANA distributions of the study groups (%)

	Augsburg (*n* = 139)	Bremen (*n* = 241)	Essen (*n* = 282)	Hamburg (*n* = 238)	Hannover (*n* = 106)	Heidelberg (*n* = 193)	*p* value^a^	Total (*n* = 1199)
Sex							0.168	
Male	48.2	45.2	39.1	47.9	47.2	38.9		43.8
Female	51.8	54.8	60.9	52.1	52.8	61.1		56.2
Median age (IQR)	55 (47–63)	54 (41–62)	41 (34–50)	48 (30–62)	52 (43–62)	45 (31–59)	<0.0001^b^	49 (38–60)
Country of birth							<0.0001	
Germany	83.5	83.8	21.3	88.7	87.7	40.9		64.0
Other	16.5	16.3	78.7	11.3	12.3	59.1		36.0
BMI							<0.0001	
Underweight (<18.50 kg/m^2^)	0	1.2	0.4	1.7	2.8	2.6		1.3
Normal weight (18.50–24.99 kg/m^2^)	33.8	43.2	18.5	51.1	46.2	46.9		38.7
Overweight (25.00–29.99 kg/m^2^)	46.8	39.0	39.9	32.5	35.8	31.3		37.3
Obesity (≥30.00 kg/m^2^)	19.4	16.6	41.3	14.8	15.1	19.3		22.7
Heart attack							0.18	
Yes	5.0	0.8	2.3	1.7	2.8	2.6		2.3
No	95.0	99.2	97.7	98.3	97.2	97.4		97.7
Diabetes							0.40	
Yes	7.2	5.4	9.9	5.9	4.7	6.8		6.8
No	92.8	94.6	90.1	94.1	95.3	93.2		93.2
ANA positivity							0.742	
Negative (<1:80)	67.6	63.9	66.8	65.1	71.7	68.8		66.7
Positive (≥1:80)	32.4	36.1	33.2	34.9	28.3	31.3		33.3
ANA positivity							0.157	
Negative (<1:80)	67.6	63.9	66.8	65.1	71.7	68.8		66.7
Weakly positive (1:80 & 1:160)	25.2	29.0	29.6	32.4	26.4	25.5		28.6
Moderately positive (1:320 & 1:640)	3.6	5.8	2.5	1.3	1.9	4.7		3.3
Strongly positive (≥1:1280)	3.6	1.2	1.1	1.3	0	1.0		1.3

*ANA* anti-nuclear autoantibody, *IQR* interquartile range, *BMI* body mass index

^a^Chi-square test or Fisher’s exact test for differences across study centers

^b^Kruskal–Wallis test for differences across study centers

### Sex and age-specific ANA prevalence rates

**Fig. 1:**
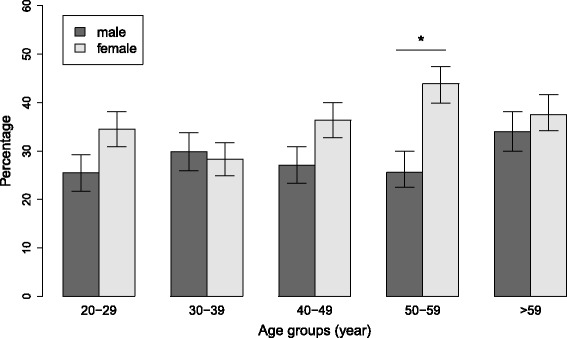
ANA positivity by sex and age groups. ANA positivity was defined as a titer ≥ 1:80. *Whiskers* indicate 95% confidence intervals. *p = 0.003

**Fig. 2:**
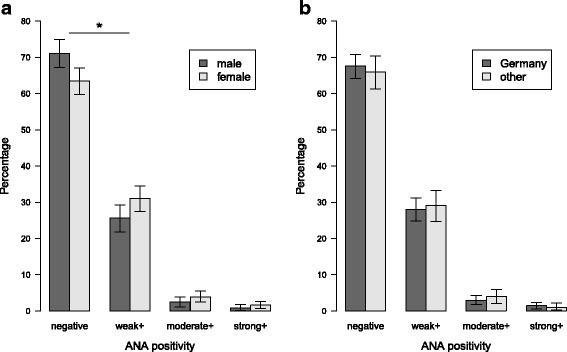
Proportion of weakly, moderately and strongly positive ANA titers by sex (**a**) and by country of birth (**b**) (*weak*, 1:80 or 1:160; *moderate*, 1:320 or 1:640; *strong*, ≥1:1280). Difference in ANA positivity by sex was significant (χ^2^ = 8.859, df = 3, *p* = 0.03). *p = 0.02. Difference in ANA positivity by country of birth was not significant (χ^2^ = 1.121, df = 3, *p* = 0.77). *Whiskers* indicate 95% confidence intervals. *ANA* anti-nuclear autoantibody

The proportion of participants with a positive ANA test (titer ≥ 1:80) was 33.3% (95% confidence intervals: 30.7–35.9%). There was a trend toward more frequent ANA positivity with increasing age among women (χ^2^ = 6.983, df = 4, *p* for trend = 0.09) but not men. With the exception of the age group 30–39, the proportion of ANA positivity was higher among women than men in all age groups, with the most significant difference in the age group 50–59 (Fig. 1). There was no significant difference in the proportion of positive samples across study centers (Table 1, χ^2^ = 2.727, df = 5, *p* = 0.742). Of the 1196 participants with available ANA results (measurements could not be obtained from three samples), 342 (28.6%), 40 (3.3%) and 16 (1.3%) were classified as weakly, moderately and strongly positive, respectively. The prevalence of weakly, moderately and strongly positive ANA titers was higher among women than men (Fig. 2a, χ^2^ = 8.859, df = 3, *p* = 0.03). There were no differences in ANA positivity between participants born in Germany and abroad (Fig. 2b).

### ANA staining patterns

**Fig. 3:**
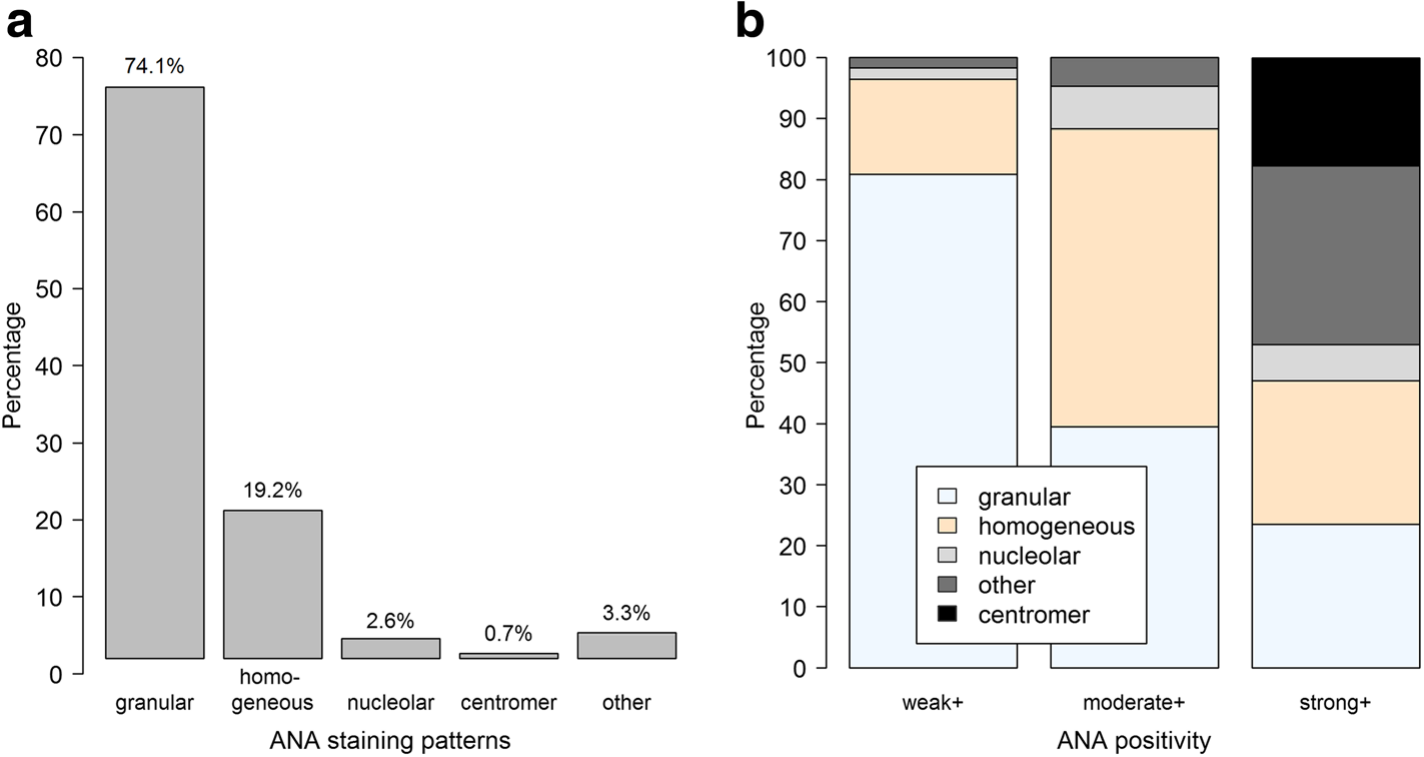
ANA staining patterns. Frequencies of patterns across all samples (**a**) and according to degree of positivity (**b**) (*weak*, 1:80 or 1:160; *moderate*, 1:320 or 1:640; *strong*, ≥1:1280). *ANA* anti-nuclear autoantibody

The most frequent ANA staining patterns were granular/fine granular pattern (74.1%), followed by homogeneous/homogeneous fine granular (19.2%) and nucleolar (2.6%) (Fig. 3a). The granular/fine granular pattern predominated in samples with weak ANA positivity (Fig. 3b). The centromere pattern was only present among strongly ANA-positive samples (Fig. 3b).

### Second-step autoantibody detection

The results of confirmatory testing for 19 specific autoantibodies are presented in Table 2. Of the 10 (53%) detected autoantibodies, anti-DFS70 (*n* = 13) and anti-dsDNA (*n* = 7) were the most frequently detected. There was a tendency for more frequent positivity for second-step autoantibodies with increasing ANA titer (χ^2^ = 3.072, df = 1, *n* = 83, *p* for trend = 0.08).

**Table 2:** Proportion of positive results for specific auto-antibodies from confirmatory assays (%)

			Number and proportion of positive results in samples with ANA titer (*n* (%))		
Antibody	Number of positive results (*n*)	Proportion of positive results for all antibodies (%) (*N* = 83)	1:80 and 1:160	1:320 and 1:640	≥1:1280
DFS-70	13	15.7	3 (23)	6 (46)	4 (31)
dsDNA	7	8.4	2 (29)	5 (71)	0 (0)
SSA	4	4.8	0 (0)	2 (50)	2 (50)
Nukl	4	4.8	1 (25)	3 (75)	0 (0)
Histon	4	4.8	0 (0)	4 (100)	0 (0)
AMA	2	2.4	0 (0)	0 (0)	2 (100)
CENPB	2	2.4	0 (0)	0 (0)	2 (100)
ENA	2	2.4	0 (0)	2 (100)	0 (0)
sp100	2	2.4	0 (0)	0 (0)	2 (100)
SSB	1	1.2	0 (0)	1 (100)	0 (0)
Myositis blot	0	0	0 (0)	0 (0)	0 (0)
SSC blot	0	0	0 (0)	0 (0)	0 (0)
Rib-P	0	0	0 (0)	0 (0)	0 (0)
Jo-1	0	0	0 (0)	0 (0)	0 (0)
U1 RNP	0	0	0 (0)	0 (0)	0 (0)
Sm	0	0	0 (0)	0 (0)	0 (0)
Gp210	0	0	0 (0)	0 (0)	0 (0)
LBR	0	0	0 (0)	0 (0)	0 (0)
F-actin	0	0	0 (0)	0 (0)	0 (0)

### Association between ANA and cardiovascular and metabolic disorders

Table 3 presents age and sex-adjusted ORs for the cardiovascular and metabolic parameters assessed. There was no association between ANA positivity and the self-reported (diabetes, elevated blood cholesterol/lipids) or objectively measured (obesity, hypertension) parameters. Likewise, there were no associations between ANA staining patterns and these variables, even when only individuals with titers ≥ 1:80 were considered (data not shown). An association with heart attack could not be tested due to the low number of reported cases (Table 1).

**Table 3:** Age and sex-adjusted odds ratios for the cardiovascular and metabolic disorders assessed (results of the four mixed-effects logistic regression models)

	Obesity^a^		Diabetes^b^		Hypertension^a^		Elevated blood cholesterol and/or lipids^b^	
	AOR (95% CI)	*p* value^c^	AOR (95% CI)	*p* value^c^	AOR (95% CI)	*p* value^c^	AOR (95% CI)	*p* value^c^
ANA positivity		0.99		0.73		0.66		0.71
Negative (*n* = 756)	Reference		Reference		Reference		Reference	
Weakly positive (*n* = 320)	0.98 (0.71–1.35)		0.86 (0.49–1.50)		0.72 (0.47–1.10)		1.13 (0.83–1.53)	
Moderately or strongly positive (*n* = 53)	1.00 (0.50–1.97)		0.66 (0.19–2.28)		1.00 (0.45–2.23)		0.94 (0.50–1.77)	

*ANA* anti-nuclear autoantibody, *AOR* adjusted odds ratio, *BMI* body mass index, *CI* confidence interval

^a^Objectively measured weight and height and blood pressure (see Methods). Obesity was defined as BMI ≥ 30 kg/m^2^. Hypertension was defined as a systolic or diastolic blood pressure ≥ 140 mmHg and/or 90 mmHg, respectively

^b^Self-reported information

^c^Tests of fixed effects

## Discussion

Using a semi-automated high-throughput system, we determined the frequencies of ANAs in the German general population at different titer cutoff point and patterns and looked for associations with selected cardiovascular and metabolism-related diseases. The detected prevalences were comparable with published values from other studies, but we did not detect any associations between ANAs at any titer or pattern and the cardiovascular and metabolic diseases assessed.

Only a few studies on ANA prevalence have so far been conducted among the general population [5, 6, 19]. None of them used population-based sampling as we did. A Mexican study involving healthy individuals (blood donors, health care workers and relatives of patients with connective tissue disorders) demonstrated similar prevalence rates; for example, an ANA positivity of 35% with a titer ≥ 1:40 [6]. Guo et al. [5] used a titer cutoff point of 1:320 to determine ANA positivity among healthy individuals in China and showed that around 6% of participants were positive. This figure was comparable with our study. Andersen-Ranberg et al. [20] examined the prevalence of nonorgan-specific autoantibodies among Danish healthy centenarians and observed a slightly higher ANA prevalence (37%, titer ≥ 1:40) than in our study. The international recommendation for the determination of ANA indicates titers of 1:160 or above as positive [21], and the European Autoimmunity Standardization Initiative (EASI) recommends sera with titers of 1:80 as borderline and with titers ≥ 1:160 to be considered positive [22]. The aim of both initiatives was to improve the diagnostics of patients with systemic autoimmune rheumatic diseases, but not the examination of the prevalence of ANA in the general population. Therefore, as suggested by the manufacturer of the HEp-2-cell assay, in our study we used a titer ≥ 1:80 to define sera as positive. This cutoff point represents a sensitivity of 98.1% and a specificity of 91.2% of the AKLIDES system for the detection of ANAs in 156 sera with defined antinuclear antibodies and 263 nonselected blood donors [23]. Low ANA titers may not be of clinical significance [1], but higher titers might predict the development of autoimmune diseases such as SLE. Arbuckle et al. [24] investigated the onset of ANAs before diagnosis of SLE and observed that ANAs (with a dilution of 1:120) were present in 78% of SLE patients before diagnosis. We found about 3.3% and 1.3% of our participants to have moderately (titer of 1:320 or 1:640) and strongly (≥1:1280) positive titers, respectively. These individuals may be at higher risk of developing autoimmune diseases.

Why did our study not detect any association between ANA titer and/or pattern and the selected cardiovascular and metabolic variables? One reason may lie in the inherent methodological differences from other studies on the topic (summarized in Table 4). In contrast to those studies, which used diagnoses from medical records and were based on physical examinations, our analysis was based in part on participants’ self-reported information regarding diabetes and elevated blood cholesterol (see Methods). However, the lack of associations between ANAs and BMI or hypertension was determined based on physical measurements obtained in the study centers. This finding agrees with the lack of association with hypertension reported by Ishikawa et al. [25], but also suggests that the association with BMI in women reported by Gonzalez et al. [10] may not apply across populations.

**Table 4:** Summary of publications reporting associations between ANAs and various cardiovascular and metabolic disorders and death

Author/year	Country	Study design	Sample size	Participants’ age, mean ± SD	Outcome	Method used to measure the outcome	Main findings
Sedaghat et al. 2014 [3]	Iran	Patient-based	140	56.4 ± 10.8	Ischemic heart disease, comparison of ANA positivity between patients with acute coronary syndrome and chronic stable angina	Coronary angiography	ANA positivity higher in patients with chronic stable angina; association with severity of coronary stenotic lesions
Chou et al. 2011 [4]	China	Patient-based	13,345	11.4 ± 5.0	Risk of death	National Death Registry	High titer of ANAs associated with increased risk of death
Heras et al. 2010 [11]	Greece	Patient-based	70 (type 1 diabetes) 28 (type 2 diabetes) 20 (control)	34.0 ± 9.1 64.0 ± 9.5 45.0 ± 16.2	Diabetes	Not mentioned	ANA positivity higher in type 1 diabetes than in healthy individuals
Gonzalez et al. 2008 [10]	Canary Islands	Community-based	702	Not reported	Obesity	Anthropometric measurements (BMI, waist circumference, waist/height ratio)	Inverse association with obesity in women, no association in men
Ishikawa et al. 2008 [25]	Japan	Community-based	2875	63.0 ± 10.0	Microalbuminuria, BMI, diabetes, hypertension, hypercholesterolemia	Almost all outcomes measured	Bivariate analysis: no association between BMI, diabetes, hypertension and ANA positivity
Liang et al. 2009 [9]	USA	Patient-based	7852	47.5 ± 17.0	Myocardial infarction, heart failure, peripheral vascular disease and risk of death	Medical records	ANAs associated with increased risk of cardiovascular diseases and mortality

*ANA* anti-nuclear autoantibody, *BMI* body mass index

We used the AKLIDES semi-automated system because it would lend itself well to high-throughput ANA determinations of large sample numbers typical of present-day “mega cohorts” like the GNC. Hospital-based studies showed that the AKLIDES system yields comparable results to the gold standard (i.e., visual inspection and evaluation by a clinical immunologist). For instance, Bizzaro et al. [26] found that the diagnostic accuracy of the AKLIDES system for automated ANA assessment was very high (sensitivity, 97.8%). Melegari et al. [27] observed very high agreement (98.9%) between automated and visual assessments of the AKLIDES system. Our study now demonstrated the feasibility of also using the AKLIDES system in a population-based analysis. Taken together, the results support the use of this system in future large-scale population-based studies that require the high-throughput capability of this system.

Limitations of the present study include its cross-sectional nature, which precludes causal inferences. Because the overall sample size was relatively small, it may not have been sufficient to detect significant associations between ANA positivity and the metabolic outcome variables studied. Thus, further research examining these relationships is required. It would have been important to validate the clinical relevance of ANA positivity, in particular of high titer, by testing for associations with autoimmune disorders such as SLE or SjS. However, this was not possible due to the very low numbers of self-reported cases (SLE = 4; SjS = 7), which are consistent with the low prevalences of these disorders in Germany [28, 29]. We pooled data from two cross-sectional studies (i.e. pretests 1 and 2), which were conducted in 2011 and 2012. There were slight differences in the designs of the questionnaires used in the pretest 1 and 2 studies and in the methodology of the studies. For example, blood pressure was only measured once in the Essen study center, while it was measured three times in the other study centers. In terms of metabolic disorders the questions did not differ. Further supporting our use of the pooled data set, there were no differences in ANA positivity between samples from pretest 1 vs. pretest 2.

## Conclusions

The prevalence of ANA positivity in the German general population was similar to values reported from other countries. Contrary to other studies, there was no association between ANA positivity and self-reported and objectively measured selected cardiovascular and metabolic disorders.

## Abbreviations

ANA: Anti-nuclear autoantibody
BMI: Body mass index
FSU: Former Soviet Union
GNC: German National Cohort
SjS: Sjögren’s syndrome
SLE: Systemic lupus erythematosus
